# Centring adult learners and recognitive justice: work-integrated learning for internationally educated health professionals in Canada

**DOI:** 10.1007/s40955-026-00335-6

**Published:** 2026-04-21

**Authors:** Hongxia Shan, Jiin Yoo

**Affiliations:** University of British Columbia, Vancouver, BC Canada

**Keywords:** Work-integrated learning (WIL), Health education, Adult learning, Bridging programmes, Recognitive justice, Immigration and labour market integration, Work-integrated learning (WIL), Gesundheitsbildung, Erwachsenenbildung, Brückenkurse, Anerkennungsgerechtigkeit, Migration und Arbeitsmarktintegration

## Abstract

In Canada, immigrants are a new demographic in work-integrated learning, such as practicums and preceptorships. While work-integrated learning is widely acknowledged as an effective strategy for preparing students for employment, little research has examined the experiences of immigrants in WIL. This paper addresses this gap with a study of internationally educated health professionals. It highlights the benefits and challenges that internationally educated health professionals faced in work-integrated learning. This paper extends work-integrated learning scholarship by centring immigrants as diverse adult learners and recognitive justice. It emphasises the need to reconceptualise work-integrated learning to better support experienced immigrant professionals in accessing and integrating into regulated professions.

## Abstract (de)

In Kanada stellen Zugewanderte eine neue demografische Gruppe in arbeitsintegrierten Lernformaten, beispielsweise Praktika oder Praxisanleitungen, dar. Obwohl arbeitsintegrierte Lernformate als wirksame Strategie zur Arbeitsmarktvorbereitung gilt, wurden die Erfahrungen von Einwanderern in arbeitsintegrierten Lernformaten bislang kaum erforscht. Dieser Beitrag adressiert diese Forschungslücke durch eine Studie zu international ausgebildeten Gesundheitsfachkräften und beleuchtet Vorteile sowie Herausforderungen in arbeitsintegrierten Lernformaten. Der Artikel erweitert die Forschung, indem er Zugewandertework-integrated learning als diverse erwachsene Lernende sowie das Konzept der Anerkennungsgerechtigkeit zentriert. Er unterstreicht die Notwendigkeit, arbeitsintegrierte Lernformate neu zu konzipieren, um erfahrene Fachkräfte beim Zugang zu reglementierten Berufen und ihrer beruflichen Integration besser zu unterstützen.

## Introduction

Work-integrated learning (WIL) combines academic study with hands-on experience in the workplace and is globally promoted as an effective means of preparing higher education students for the labour market (Bowen and Drysdale 2017; Raza and Chua 2025). As an umbrella concept, WIL encompasses a range of training and educational models, including but not limited to cooperative education, industry-engaged learning, internships, practicums, clinical placements, fieldwork, preceptorships, service learning, workplace learning and so on (Stirling et al. 2024). WIL can be implemented at different scales. It can be embedded within a course, serve as a central component of a programme, or exist as a stand-alone programme. Students may or may not be compensated for their work, depending upon the programme model, academic discipline, or local and national contexts (Zegwaard and Pretti 2023). For example, whereas cooperative education is traditionally paid, practicums and service-learning are often unpaid. While WIL models vary, they all typically offer structured opportunities for learners to apply knowledge in real-world contexts, gain work experience, build social networks, and develop practical skills. They are also known for enhancing employability, supporting personal growth, and fostering professional identity formation (Raza and Chua 2025; Zegwaard and Pretti 2023).

In Canada, immigrants have emerged as a new demographic in WIL-embedded programmes. Based on 2016 Wave 3 of the Longitudinal and International Study of Adults (LISA), Iacampo (2023) reported that 11% of immigrant students in higher education took part in WIL. This trend is likely driven by the need for immigrants to have their prior qualifications recognised in the host labour market, a persistent challenge facing newcomers in Canada and other immigrant-receiving countries (e.g., Baxter 2025; OECD 2025). To become recognised, immigrants, particularly those highly educated, tend to pursue further education as a pathway to employment (e.g., Adamuti-Trache 2016). The growing presence of migrant students on Canadian campuses is also shaped by the country’s two-step immigration pathway, which attracts international students and facilitates their transition to permanent residents (Schinnerl and Ellermann 2023).

The WIL opportunities immigrants access are not limited to degree or diploma programmes. Recently, universities and colleges, in partnership with professional regulators, employers, and other stakeholders, have been involved in designing or delivering retraining and bridging programmes to facilitate immigrants’ licensure process (e.g., Neiterman et al. 2018; Sattler et al. 2015), and to address skill gaps in specific industries (e.g., Sadiq et al. 2025). These programmes are typically designed to help immigrants requalify to practice in designated, especially regulated professions. Practicum and workplace placement are frequently a core component of these programmes. Retraining and bridging programmes for immigrants have been the subject of evaluation and research (e.g., Covell et al. 2018), but limited attention has been paid to these programmes from a WIL perspective.

This paper addresses the limited understanding of immigrants’ experiences of WIL. Specifically, drawing on a qualitative study of the career navigation experiences of internationally educated health professionals (IEHPs), it explores the impacts of WIL programmes on these professionals and the challenges they faced. The paper begins by setting the context of the research, before introducing WIL as a conceptual framework and expanding it by centring adult learners and recognitive justice. It then presents the research methods and research findings, followed by a discussion of the significance and implications of the findings. The paper concludes by recapitulating its contributions and limitations.

## Context of the study

To address healthcare labour shortages, the Canadian government actively recruits healthcare workers from abroad (Government of Canada 2023). However, skilled immigrants, especially those arriving directly from non-Western countries, continue facing barriers to employment, which include but are not limited to a lack of familiarity with Canadian systems and workplace culture, language and communication barriers, and a lack of social network (Crea-Arsenio et al. 2022). On top of all these, recognition remains a persistent issue (Baxter 2025). For people seeking entry to regulated professions, such as those in the healthcare sector, the issue of recognition is complex as the provincial and territorial governments manage professional regulation, each having its distinct requirements. Prejudice, discrimination, and employers’ expectations of Canadian work experience further complicate recognition, often leading to immigrants being misrecognised and marginalised in the labour market (Al-Btoush and El-Bcheraoui 2024; Esses 2021).

Canada has taken steps to improve recognition of foreign qualifications by introducing legislative and policy changes aimed at reducing barriers for internationally educated professionals. Ontario (ON) led early efforts with *the Fair Access to Regulated Professions Act* in 2006. British Columbia (BC) introduced *the International Credentials Recognition Act* in 2023 to streamline credential recognition practices across regulated professions. To support immigrants’ integration into their fields of training, services and training programmes have been developed. In the healthcare sector, for example, international medical graduates (IMGs) may undertake training prior to the residency required of all medical graduate students. Other IEHPs who do not fully meet licensure requirements may also be asked to undertake bridging programmes (Connelly et al. 2023). These training programmes are typically offered through higher education institutions.

Research on immigrant retraining and bridging programmes shows that these programmes help close credential and language gaps, expand professional networks, and accelerate labour market entry (Sattler et al. 2015). Meanwhile, immigrants also reported problems such as programmes’ misalignment with IEHPs’ needs, limited clinical placements, and financial sustainability (Neiterman et al. 2018). Studies on bridging programmes for internationally educated nurses (IENs) also highlight positive program effects: enhanced understanding of the Canadian healthcare system, clearer professional roles, stronger nursing competencies, and improved occupation-specific language and communication skills (e.g., Cubelo et al. 2025).

Notably, these programmes typically involve opportunities for immigrants to gather Canadian work experience through the incorporation of clinical placements, preceptorships, simulated practices etc. While existing studies recognise the importance of such work-based learning in facilitating immigrants’ integration into the Canadian workforce (Covell et al. 2018), rarely has research addressed these programmes from a WIL perspective.

## Centring adult learners: work-integrated learning reconceptualised part I

Rooted in experiential learning and a constructionism, WIL places a clear emphasis on authentic experiences (McRae and Johnston 2016). It values what Kolb and Kolb (2018) describe as “deep experiencing” (p. 9), moments that interrupt the normal flow of life and confront individuals with unfamiliar situations or challenges. Such experiences may elicit reflective observation, abstract conceptualisation, and experimentation, leading to “deep learning” (Kolb and Kolb 2018, p. 10), which can be both cognitive and affective (Matsuo and Nagata 2020). Various WIL models have been developed to guide higher education institutions in the implementation, tracking, and evaluation of such initiatives. Attention is typically focused on the attributes and parameters of the programmes that may turn real-life work experiences into positive learning outcomes for WIL participants (e.g., Moore 2010; Stirling et al. 2024). Participants in these programmes are typically understood to be young graduates entering the labour market for the first time. However, as Billett and Choy (2011) point out, WIL can support individual development across working life.

Understanding WIL for immigrants requires recognising adult learners as distinct from younger learners. In this regard, Andragogy (Knowles et al. 2014) highlights adults bring extensive experience, which constitute a rich resource for learning and the core of their self-identity. Further, adults’ orientation of learning is life-centred, driven by tasks and problems in real-life situations. Their readiness to learn arises from immediate needs or interests. Adults are also characterised by a strong desire for self-esteem, self-growth, and a distinct psychological need for autonomy and self-direction. With these assumptions, Andragogical approaches advocate that learners be involved in identifying learning needs, formulating goals, gathering resources, choosing and implementing strategies, and evaluating outcomes. Amid postmodern complexity and uncertainty, Hase and Kenyon (2000) extend Andragogy into Heutagogy, arguing that adult education should prepare individuals to become capable people who know how to learn. To do so, learners should determine their learning pathways while navigating evolving complex work contexts, with educators playing the role of co-learners, supporters, and facilitators of learning design.

While foregrounding adult learners is essential in examining immigrants’ WIL experiences, it is equally important to challenge the image of a universal adult learner, conjured through Andragogy and Heutagogy, which privileges white, middle-class, male identities (Dantus 2021). The emphasis on autonomy and self-direction/determination obscures the fact that people from different social backgrounds may not have the same control over their conditions. Furthermore, while adults are distinguished by their rich experiences, not all experiences are recognised equally. Treating adult learners as a homogeneous group is problematic.

## Towards recognitive justice: work-integrated learning reconceptualised part II

Notably, recent WIL scholarship has incorporated intersectionality theories, culturally relevant pedagogy, and critical race theory to promote equity and inclusivity (McRae et al. 2023; Stirling et al. 2024). Together, this emerging body of work underscores how intersecting axes of differences, such as race, gender, and class, shape experiences of privilege and marginality and highlights the need for culturally responsive support. To address skilled immigrants at the centre of WIL, it is, however, imperative to go beyond acknowledging group-based differences and cultural accommodation. It is rather important to centre recognitive justice (e.g., Guo and Shan 2013; Shan and Guo 2013).

For Honneth (e.g., Fraser and Honneth 2003), recognition is a foundational condition for human flourishing and a basic human need essential to identity formation and self-realisation. As individuals come to understand themselves through their relations with others, misrecognition, i.e., the denial or distortion of recognition, undermines an individual’s self-confidence and self-esteem and can result in psychological harm (Fraser and Honneth 2003). The normative cultural order of recognition, according to Honneth, generates not only moral and social struggles but also struggles that take economic and distributive forms (Fraser and Honneth 2003). In the realm of knowledge, Miranda Fricker (2007) advances the concept of epistemic injustice. She argues, misrecognition may not be intentional. It is, however, a form of discrimination against people, particularly outsiders, in their capacities as knowers. Such discrimination is sustained through testimonial injustice and hermeneutic injustice. Testimonial injustice occurs when a speaker’s contribution to collective knowledge through communicative exchanges is accorded less credibility due to prejudice on the part of the hearers. Hermeneutic injustice arises when gaps in collective interpretive frameworks render certain social experiences unintelligible or difficult to articulate.

Nancy Fraser (Fraser and Honneth 2003) approaches misrecognition as a form of injustice stemming from institutionalised hierarchies of cultural value. Cultural misrecognition intersects with economic maldistribution. Together, they deprive marginalised groups of the parity of participation across different realms of life. Fraser’s tripartite social justice hence comprises economic redistribution, political representation, and cultural representation. Her recognitive justice is about the elimination of institutionalised hierarchies of value that misrecognise cultural groups, keeping them from equitable participation in social and economic life. Notably, recognitive injustice is linked not only to cultural differences but also to historical and global geopolitical power struggles that shape the epistemic hierarchy that centres Western knowledge and scientific rationality. Decolonial and postcolonial scholars highlight the concept of “epistemic violence” (Spivak 1988, p. 24) to draw attention to how dominant, often Western-centric, knowledge systems silence, suppress, and devalue other ways of knowing and being, and force cognitive assimilation (e.g., de Sousa Santos 2014). As an antidote, they advocate, for instance, for an “ecology of knowledges” (de Sousa Santos 2014, p. 188), emphasising the need for meaningful dialogue among different knowledge systems.

If immigrants are recognised as diverse adult learners and recognitive justice is foregrounded, WIL can be transformed into a dynamic space for engagement. In this space, immigrants should be able to articulate differences, exercise autonomy and self-direction and participate in the making of their learning trajectories. WIL must attend to the multiplicity of identities, perspectives and ways of knowing. Such an approach means that WIL needs not only to facilitate how individuals may negotiate identities and roles within an unfamiliar cultural and institutional framework, but also to foster dialogues that confront misrecognition, nurture mutual understanding, and create opportunities for more equitable participation. Ultimately, WIL must become a space where difference, power, and possibility are continually explored, interrogated, and negotiated.

## Research methods

Empirically, this paper draws on a study of the career pathways of IEHPs in Canada, conducted between 2023 and 2024. The study asks two main research questions. First, how do IEHPs navigate differences and build their career pathways within the healthcare professions? Second, what social processes and practices facilitate or hinder their career-building processes, and in what ways? The study involves career-history interviews with 22 IEHPs, 10 in British Columbia and 12 in Ontario. All respondents were recruited through social media platforms and advertisement flyers distributed via health-related organisations in the two provinces. Respondents immigrated to Canada in their adult years from Mainland China, Hong Kong, South Korea, India, Jordan, the Philippines, and Japan. They all received at least a degree in healthcare or a related field from outside Canada. Each interview lasted approximately 90 min and was conducted either over Zoom or in person. Interviews were recorded with the consent of each participant, transcribed verbatim, and then sent to participants for member-check.

Half of the respondents (11) participated in WIL. Four of them (A) went through bridging or retraining programmes as requested by regulatory bodies as a condition for qualifying for professional licensure. These programmes involved both in-class learning and supervised clinical placements or practicum. Seven of them completed WIL while registered in a diploma or degree programme in Canada, four as international students (B) and three as permanent residents (C). Such experiences included preceptorships, practicums, and simulated or laboratory-based training (see Table 1).

**Table 1:** Demographic Profile

Pseudonym	Gender	Age	Ethnicity	Year of landing	Prior profession	Current profession	Work-Integrated Learning
*A. Participants in bridging or retraining programs as part of professional requalification*							
Jihee	Female	46–55	South Korean	2013	Registered Nurse	Registered Nurse	Bridging Program
Clara	Female	36–45	South Korean	2015	Pharmacist	Pharmacist	Bridging Program
Safiya	Female	26–35	Jordanian	2018	Clinical Researcher	Pharmacist	Bridging Program
James	Male	66+	Hong Kong Chinese	1995	Physician	Physician	Retraining Program
*B. Participants in diploma or degree programs as international students*							
Wallis	Female	26–35	Hong Kong Chinese	2021	Laboratory Technologist	Student	Two-year Diploma (Pharmacy Technician)
Emma	Female	26–35	Hong Kong Chinese	2022	Registered Nurse	Student	Master (Public Health)
Hayoung	Female	36–45	South Korean	2021	Registered Nurse	Registered Nurse	Post Graduate Certificate (Nursing Practice)
Bella	Female	36–45	Indian	2015	Registered Nurse	Registered Nurse	Graduate Certificate (Advanced Health Care Leadership)
*C. Participants in diploma or degree programs as permanent residents*							
Madeline	Female	46–55	Mainland Chinese	1998	Pharmacist	Registered Nurse	Diploma (Nursing)
Jing	Female	56–65	Mainland Chinese	2004	Physician	Cytologist	Graduate Diploma (Cytologist)
Jialin	Female	46–55	Mainland Chinese	2004	Sonographer	Sonographer	Diploma (Diagnostic Medical Sonography)

Data collected were analysed thematically (Braun and Clarke 2019). The research team inductively generated initial codes, which were then systematically compared and collated to construct themes, with the research questions in view. The analytic process was iterative and reflexive, involving ongoing team dialogue and critical interrogation of interpretations. The themes that emerged from respondents’ WIL experiences are presented in the findings section below.

## Research findings

Respondents reported both *benefits* gained from their participation in WIL programmes, and the *challenges *they encountered. This section presents both.

### Benefits

Consistent with research on WIL programmes in general (e.g., Raza and Chua 2025) and immigrant bridging programmes in particular (e.g., Covell et al. 2018; Cubelo et al. 2025), the study found that WIL helped respondents identify and bridge gaps in professional knowledge, gain locally relevant skills and competencies, and build stronger professional networks, communities, and opportunities.

#### Bridging gaps and differences in professional knowledge

While engaged in WIL, respondents identified and learned about institutional, cultural, and professional differences between Canada and their home countries (e.g., Covell et al. 2018). Such differences ranged from terminology and types of medications, and scopes of responsibilities, to broader values and approaches to healthcare practices. To give a few examples, Clara shared that the bridging programme for pharmacists helped her become familiar with medications used in Canada. Jialin, who enrolled in a diploma programme in diagnostic medical sonography, stated that during practicum, she expanded her English medical terminology, changed her communication style, and improved her confidence and comfort interacting with patients and colleagues in Canadian clinical settings.

Respondents also reported developing a deeper and more systemic understanding of Canadian healthcare practices and systems, often through comparison. Jihee, for instance, shared that during her nursing practicum at an intensive care unit (ICU) in BC, she noted that Canadian nurses had greater autonomy and discretion at work than nurses in South Korea, where nurses typically followed doctors’ instructions closely at her time. Madeline, formerly a pharmacist in Mainland China, switched to nursing by completing a diploma programme. She shared that when participating in community placements, she was exposed to “community health, palliative health … disease prevention and chronic disease, management, health promotion and end of life care,” which gave her a sense of the “true meaning of [the] healthcare system.”

#### Gaining locally relevant skills and competencies

In addition to expanded knowledge, respondents reported that WIL helped them adapt to local practices, primarily through direct engagement in real or simulated work environments (e.g., Stirling et al. 2024). In the healthcare sector, professional competency is understood as a holistic synthesis of skills, knowledge, attitudes, and values that drives high-level performance in clinical roles (Karami et al. 2017). Importantly, healthcare professions are practice-based, and professional skills and competencies are developed and demonstrated through hands-on engagement in practice. Jing described her WIL experiences as part of a cytologist diploma programme:

“Every day, we just screen … a slide first, then the teacher [read preceptor] will check. Because we have to be 97% accurate … [T]hey [expect] … not only the quality, [but] also quantity … After graduating, you … can work right away. That’s the purpose of the training.”

Similarly, Jihee emphasised that she developed practical skills and professional competencies mainly by working directly with doctors, patients, and nurses in real workplace settings. Practicums, she noted, were particularly effective in helping her consolidate and apply nursing knowledge. Her practicum experiences also prompted her to critically reflect on nursing education in South Korea. She subsequently became a keen advocate of experiential pedagogies, promoting them among former colleagues who are now nursing educators in South Korea.

Among all the skills respondents noted, communication was mentioned the most frequently. In all cases, though, communication is far from a linguistic matter; it was always discussed in conjunction with work and professional practices in Canada. Clara explained that during her practicum, she had to make deliberate efforts to learn to read handwritten prescriptions; when working in South Korea, she was used to a computer-based system. Jialin noted that her sonography practicum provided her with opportunities to engage with patients and work alongside other healthcare professionals. She believed these experiences strengthened her communication skills and eased her transition into the Canadian healthcare system.

#### Developing professional communities, networks, and opportunities

Another benefit of WIL programmes is the expansion of networks and employment opportunities (e.g., Zegwaard and Pretti 2023). In our study, respondents reported that WIL experiences helped them build professional connections and employment opportunities, and form communities that extended beyond the duration of the programmes.

Jihee completed a bridging programme for internationally educated nurses, as required by the regulator, which included practicum placements. Through these placements, she earned the trust and recognition of a manager:

“My manager just told me if you are really interested in working in [the] hospital, in emergency, just call me after your examination. So, my manager just gave me her business card. After passing the NCLEX exam [the nursing qualification exam], I just called my manager in [the]hospital. I didn’t do any interviews … I [was] just hired.”

Similarly, Clara was offered a job at the pharmacy where she completed her practicum. In both cases, through the WIL, the respondents expanded their social capital and networks and *earned* recognition by key gatekeepers.

In some cases, the networks the respondents built developed into lasting learning communities. James was required to complete a retraining programme for international medical graduates (IMGs). There he formed a study group with other IMGs of similar backgrounds, and the group remained active beyond the programme. He said: “So, we formed groups … We share our knowledge, our experience, how we deal with some typical patients … Even doctors, we have different approaches. And then we can learn from other people’s experiences.” James’s case may not be typical. It, however, suggests that beyond bridging individual IEHPs to employment, WIL-embedded programmes can also lead to the formation of learning communities that facilitate knowledge exchange, peer support, and collaborative learning (e.g., Sattler et al. 2015).

### Challenges

Respondents also reported challenges with their programmes. Some noted that the programmes did not address their professional needs at their stage of career development. Some felt their knowledge and skills were under-recognised or underutilised. A few also described experiences of being othered and encountering workplace discrimination.

#### Misalignment with immigrants’ professional needs

Several respondents reported that the programmes they attended did not address their professional needs or take into account their stage of career lives. This is particularly true for the four respondents who were required to complete bridging or retraining programmes to become licensed.

James immigrated to Canada in 1995. Despite 20 years of experience as an orthopedic surgeon in Hong Kong and a UK fellowship, he was required to complete a two-year retraining programme for IMGs, followed by another two-year family medicine residency. When asked why he did not return to orthopedics, he explained that becoming licensed as a specialist would have required a five-year residency. He also suggested that his peer immigrants faced the same issue and settled on practicing as family doctors. He considered the retention program unusually lengthy and suggested replacing it with a structured mentorship program.

Clara and Safiya completed a bridging programme for pharmacists in BC and ON, respectively. Clara felt that her bridging programme relied too heavily on lectures and covered content with which she was already familiar. As an experienced pharmacist, she found the emphasis on theoretical knowledge and memorisation-based testing redundant and wished the experiential component had been longer. Safiya voiced a similar critique. She shared that her programme was dominated by standard lectures, assignments, and exams. She noted that the programme resembled undergraduate coursework, rather than specialised training designed for internationally trained professionals who already possess substantial knowledge and experience.

Respondents enrolled in diploma programmes also pinpointed mismatches between programme structures and their professional needs. Wallis, a former laboratory scientist, enrolled in a pharmacy technician programme in ON. She questioned the relevance of the required English courses:

“That’s redundant, for sure. But you can’t escape it, because it’s part of [the] curriculum … It’s about how to critique an essay, which I think is useless, because it’s not [even] about academic writing, it teaches you how to do citation …”

Wallis’ frustration reflects a broader concern among the research respondents: the disconnect between IEHPs’ actual language needs for work and the way English is taught in post-secondary settings. Several respondents, including those who passed the English exams as part of their licensure requirements, stressed that test success did not equate to readiness for workplace communication.

#### Under-recognised and under-utilised IEHP knowledge and skills

In addition to unmet professional needs, most respondents reported a lack of recognition of their prior knowledge and skills in the programmes they joined.

Emma, pursuing a master’s degree in public health, described her classmates’ mistrust of her prior knowledge.

“[T]he medication we use in Hong Kong and here is … different. We may use different kinds of painkiller … here, they … just use like, the Advil, Tylenol … I recommend[ed] different medications to my classmates, and they don’t trust me.”

Emma’s experiences exemplify Fricker’s (2007) testimonial injustice. While her peers may not have intentionally dismissed her, deep-seated biases against outsiders could have unconsciously led them to devalue the knowledge that she tried to communicate and contribute.

The issue of recognition is not only interpersonal but also institutional. Clara recalled that while she excelled at school in South Korea, she initially failed a course in the bridging programme. The problem was that in a mock test, she omitted one side effect that she believed had an extremely low chance of occurring, given her years of professional experience. She felt she was given low scores because the assessment mechanisms did not allow for professional judgment informed by her prior experiences.

Under-recognition of immigrants’ prior experiences is particularly an issue for immigrants undergoing a downward career transition, leading to a narrowing of their scope of responsibilities. Wallis shared her experience:

“So technically, you can’t recommend any clinical stuff [as a pharmacy technician]. Let’s say you know Tylenol [as a] painkiller … as a registered pharmacy technician, you can’t tell a patient that, even though you know the pharmacist is gonna tell them to take Tylenol, you just can’t say that. So, if you say it during the exam, then you probably fail the exam.”

While maintaining professional boundaries is important, these examples reveal a deep desire among IEHPs for meaningful recognition of their prior knowledge and experiences within Canada’s healthcare system. However, the epistemic structures of different healthcare professions may have failed to accommodate and fully leverage immigrants’ experiences and expertise, which constitute what Fricker (2007) terms hermeneutic injustice.

#### Experiences of being othered and workplace discrimination

A few respondents also reported experiences of being othered and facing discrimination.

Emma enrolled in a Master of Public Health programme. She shared her discomfort in informal workplace interactions:

“My English is not that good … I find it is really difficult to do small talks in English, especially like … When I say something, … people will just like, we will just have a dead air. That’s a little bit … (laugh) Yeah, I’m still getting used to it.”

Emma’s account exemplifies the experience of ‘othering’ reported by the respondents. Notably, respondents often framed the issue as a personal shortcoming, i.e., her struggle with English, rather than a failure of the colleagues to be inclusive. This tendency to internalise blame illustrates the psychological harm the lack of recognition may inflict, including diminished confidence and self-disparagement (Fraser and Honneth 2003).

Some respondents reported observing and experiencing workplace discrimination. Safiya was deeply disheartened by the treatment of international pharmacy graduates (IPGs) and herself by practicing pharmacists during her work placement. She compared the experiences of an international pharmacy student and a local pharmacy student this way:

“The [local] student was speaking to patients, doing clinical reviews with the patients, going over their medications, their medical conditions, giving them advice, sending faxes to the doctors, giving pharmaceutical opinion, giving injections, and the IPG was doing the blister packs … To be honest, [the local student] is much more respected than me, who has studied six years of PharmD [Doctor of pharmacy].”

After repeatedly observing differential treatment of local and international pharmacy graduates, Safiya questioned why IPGs were not trusted:

“What’s the difference between this person [Canadian student] and this person [IPG]? They both have the knowledge, they both have the student licenses, they both have the scope. You see, you’re seeing the feeling? It’s so degrading for you, you feel like you’re shattered from your inside … I was lucky that I survived this feeling, but I know some of my colleagues … just switch to another profession or something like that.”

Safiya’s account illustrates both testimonial and hermeneutical injustice as articulated by Fricker (2007). It highlights how unexamined prejudice can undermine her peers’ capacity to recognise migrant professionals as credible knowers. It also reveals the limitations of the WIL structure in acknowledging and valuing the substantial professional experiences that immigrants bring.

## Discussion

Research has highlighted that WIL is valuable for higher education institutions to prepare students for the labour market (e.g., Zegwaard and Pretti 2023; Stirling et al. 2024). Yet adult immigrants, a growing demographic in WIL-embedded programmes, have received scant attention in the literature. This paper addresses this void in the literature by exploring IEHPs’ WIL experiences in Canada. Empirically, the paper is based on a thematic analysis of the experiences of eleven IEHPs who participated in retaining or bridging programmes or diploma and degree programmes that integrate WIL opportunities such as preceptorship, practicums, and simulated laboratory learning. Notably, this paper is among the first to approach immigrants’ WIL experiences with a clear recognition of immigrants as diverse adult learners and by centring issues of recognition. The findings in the paper have significant implications for the reimagination of integration and WIL in the context of migration.

To begin with, despite their diverse healthcare backgrounds, the respondents valued the opportunities WIL provided to access real or simulated healthcare practices, which would otherwise have remained out of reach. WIL clearly constitutes a unique learning mechanism for the research respondents to expand their professional knowledge, practical skills, and competencies. While participating in WIL, respondents encountered unfamiliar and sometimes challenging situations, which opened possibilities for “deep learning” (Kolb and Kolb 2018, p. 10). These encounters prompted them to identify and reflect on differences in knowledge and practices, and assess the relevance, applicability and transferability of their past experiences to the new context, and in doing so, engage in both unlearning and new learning (Matsuo and Nagata 2020). WIL also enabled respondents to establish and expand social ties and possibly social belonging, and to become embedded in the new contexts of work and life. These findings corroborate with existing studies on the benefits of WIL (e.g., Stirling et al. 2024) and bridging programmes (Covell et al. 2018; Neiterman et al. 2018). It is clear that WIL facilitated immigrants’ recontextualization of skills, professional requalification, and development of social and professional connections, all of which are critical to their access to professional practices. In countries and contexts where the integration of immigrant labour remains a challenge, WIL can be considered a promising pathway to the labour market.

Despite its promises, WIL needs to be reimagined when implemented for the integration of immigrant professionals. The study revealed several challenges faced by the respondents: the first is a misalignment between the programmes and respondents’ professional needs, which is reported, for instance, in Sattler et al.’s (2015) study of bridging programmes for IEHPs. The other two are un(der)recognition of prior learning and knowledge, and experiences of othering and discrimination, findings that are noted in other immigrant studies (e.g., Al-Btoush and El-Bcheraoui 2024; Esses 2021). All of these suggest a lack of recognition of immigrants as experienced and capable knowers, both at the interpersonal and institutional levels.

Importantly, the respondents are not neophytes entering the labour market for the first time. Rather, they demonstrated the core Andragogical assumptions about adults (Knowles et al. 2014): a high degree of readiness to learn, driven by their desire to reclaim professional identities and a practical need to transition to healthcare practices in Canada. Respondents showed a clear discernment about what learning was necessary and what was redundant. They also brought extensive prior knowledge and a strong capacity for autonomous, self-directed learning, as evidenced by their disposition toward comparative and reflective learning. Yet, in all the programmes shared by the respondents, immigrants were positioned merely as learners adapting to local practices. None reported being involved in the design of their own learning projects. Several respondents pinpointed that their programmes resembled undergraduate training for novices, rather than being designed for experienced professionals seeking requalification.

A significant limitation of the WIL-embedded programmes is their tendency to place the entire burden (of proof) on immigrants to meet the standards and practices in the host society. The evaluative frameworks in healthcare professions do not seem to allow immigrant healthcare professionals to fully articulate themselves, which constitutes an instance of hermeneutical injustice (Fricker 2007). Moreover, the study also pointed out that, as individuals, immigrants may also be discredited as knowers. In the study, a number of cases were reported where respondents’ articulation of knowledge was dismissed, a form of testimonial injustice (Fricker 2007) that can be attributed to unexamined biases and prejudices against immigrant others. Such cases often left some respondents frustrated and emotionally drained, and according to some accounts, led some immigrants to abandon their professional pursuits.

To make WIL an effective and equitable pathway for immigrant professional integration, it must centre immigrants as diverse adult learners and recognitive justice. Rather than positioning immigrants solely as learners who must adapt to dominant professional norms, WIL initiatives need to recognise them as experienced adult professionals with the capacity and right to shape their own learning trajectories. This entails involving immigrant participants in identifying learning needs, setting goals, and determining appropriate modes of learning, thereby acknowledging their prior expertise and supporting self-directed requalification. Beyond skills acquisition, WIL also holds potential as a dialogic and relational space in which diverse professional knowledges can be exchanged and valued, fostering what de Sousa Santos (2014) describes as an “ecology of knowledges.” Attending to the relational and affective dimensions of learning is particularly critical, as recognition profoundly shapes immigrants’ sense of professional self. Without intentional mechanisms to counter epistemic marginalization and deficit positioning, WIL risks reproducing misrecognition and exclusion. Centring recognition within WIL design and practice is therefore not only a matter of pedagogical effectiveness, but also of significance for social justice.

## Conclusion

This paper explored the experiences of IEHPs with WIL-embedded programmes in Canada, with a focus on the benefits they gained and challenges they faced. This research is limited in that it did not seek to distinguish among types of WIL programmes or among professions within the healthcare sector. Neither are the participants representative of all IEHPs in Canada. Despite these limitations, the findings suggest WIL may be a strategy worth considering to facilitate immigrants’ labour market integration. More importantly, the study contributes to the recent movement in WIL towards equity and inclusivity. By centring adult learners and recognitive justice, this paper offers important insights into how WIL can be transformed into an equitable pathway for immigrants seeking integration into regulated professions. To achieve this, WIL programmes must be intentionally designed to recognise immigrants’ diverse experiences as adult learners and their stages of professional lives and needs. Programmes should critically examine the epistemic frameworks of professions and challenge the deficit positioning of immigrants. A more transformative approach to WIL is to reimagine it as a pragmatic and dialogic space that fosters reciprocal knowledge exchange, mutual learning, and an appreciation of diverse forms of knowledge.
